# Downregulation of miR-139-5p contributes to the antiapoptotic effect of liraglutide on the diabetic rat pancreas and INS-1 cells by targeting IRS1

**DOI:** 10.1371/journal.pone.0173576

**Published:** 2017-03-27

**Authors:** Jin Li, Lei Su, Ying-ying Gong, Mei-lin Ding, Shu-bin Hong, Shuang Yu, Hai-peng Xiao

**Affiliations:** 1 Department of Geriatrics, the First Affiliated Hospital of Sun Yat-sen University, Guangzhou, Guangdong, P.R. China; 2 Department of Endocrinology, the First Affiliated Hospital of Sun Yat-sen University, Guangzhou, Guangdong, P.R. China; Universitat de Barcelona, SPAIN

## Abstract

Liraglutide is administered as glucagon-like peptide-1 (GLP-1) receptor agonist for diabetic patients and can protect pancreatic β-cells by inhibiting their apoptosis. MicroRNA-139-5p (miRNA-139-5p) participates in the regulation of cancer cell apoptosis. However, it is not clear whether miR-139-5p contributes to the anti-apoptotic effect of liraglutide in β-cells. The objective of the present study was to investigate the role of miR-139-5p on apoptosis of pancreatic β-cells. MicroRNA levels in pancreatic tissue from diabetic rats and INS-1 cells treated with liraglutide were measured by real-time quantitative RT-PCR. The role of miR-139-5p on apoptosis was studied by transfecting INS-1 cells with miR-139-5p mimics. The mRNA and protein expression of the target gene, insulin receptor substrate-1 (IRS1), were measured by qRT-PCR and Western blot, respectively. Apoptosis in rat pancreatic tissue and INS-1 cells was detected by TUNEL and annexin V/propidium iodide costaining. Apoptosis of pancreatic tissue from diabetic rats and INS-1 cells was decreased by administration of liraglutide. The expression of miR-139-5p increased in the pancreas of diabetic rats and decreased with liraglutide treatment. Incubation with liraglutide (100 nM) for 48 h attenuated the expression of miR-139-5p and increased the mRNA and protein levels of IRS1. Direct regulatory effects of miR-139-5p on IRS1 were found by a dual-luciferase reporter assay. Transfection of INS-1 cells with miR-139-5p mimics led to decreases in the mRNA and protein expression of IRS1. In conclusion, our observations suggest that decreased miR-139-5p expression contributes to the anti-apoptotic effect of liraglutide on the diabetic rat pancreas and INS-1 cells by targeting IRS1.

## Introduction

The worldwide prevalence of type 2 diabetes (T2DM) is dramatically increasing. It is expected that 642 million individuals will be affected with the disease by the year 2040[1]. Pancreatic β-cell dysfunction is recognized as a prerequisite for the development of T2DM. β-cells are gradually destroyed by excessive nutrients such as glucose (glucotoxicity) and free fatty acids (FFA) (lipotoxicity), resulting in β-cell failure in T2DM [2]. Therapeutic modalities that improve β-cell function are considered critical for the management of T2DM.

Glucagon-like peptide-1 (GLP-1) and its synthetic analogues reduce blood glucose by modulating glucose-dependent insulin secretion [3]. Studies using primary neonatal rat islets demonstrated that liraglutide inhibits both cytokine- and FFA-induced apoptosis via the phosphoinositide 3-kinase (PI3K)-mediated pathway [4], although the exact mechanisms have not yet been clearly demonstrated [5].

MicroRNAs (miRNAs) are a class of endogenous non-coding small RNAs expressed in eukaryotes that are generally about 19–23 nucleotides in length. MicroRNAs inhibit translation by binding to the 3′ untranslated region (3′ UTR) of their target mRNAs. Recent studies have demonstrated that miRNAs are important regulators of islet cell apoptosis, differentiation, and proliferation. Overexpression of miR-34a, miR-146a, miR199a-5p or miR-29 in MIN6 cells negatively impacts on beta cell function [6]. Overexpression of miR-132 and inhibition of miR-184 protects beta cells against palmitate- or cytokine-induced apoptosis [7]. Blocking miR-375 expression increases PDK1 protein level and glucose-stimulatory action on insulin mRNA and DNA synthesis [8, 9]. MiR-139-5p, a recognized tumor-suppressing miRNA, has been shown to be down-regulated in a variety of cancers [10, 11]. Overexpression of miR-139-5p promotes lung cancer cell apoptosis, which is associated with caspase-3 activation [12]. However, to date, there is no report investigating the role of miR-139-5p on β-cell apoptosis.

Considering the wide range of genes that are regulated by these miRNAs, we hypothesized that the anti-apoptotic effect of liraglutide on pancreatic β-cells is mediated through specific miRNAs (i.e. miR-139-5p). Moreover, we sought to explore the target genes of miR-139-5p following liraglutide treatment.

## Materials and methods

### Ethical statement

All experiments were performed in compliance with relevant Chinese and institutional laws and guidelines and were approved by the local ethics committee of Sun Yat-sen University (documentation no. 356, 2012).

### Animals

Fifty 1-week-old male Sprague Dawley (SD) rats (certification number: 4408501210) were purchased from the Laboratory Animal Center of Sun Yat-sen University (License Number: SCXK 2011–0029). All rats were housed in a specific pathogen free animal facility with a 12 h light/dark cycle and *ad libitum* access to chow and tap water. The diabetic model of SD rats was established by feeding with a high-fat diet (HFD) from 2-weeks of age and intraperitoneal injection of streptozotocin (STZ) (30 mg/kg BW) at 10-weeks of age. Rat were fed with a diet consisting of 45% calories from fat, 18% calories from protein and 37% calories from carbohydrates. Fasting glucose from tail samples was measured at 2 and 7 days after injection of STZ with a glucose meter (ACCU-CHEK Aviva, Roche) and when glucose level was over 16.7 mmol/L, rats were confirmed as diabetic. Diabetic rats were divided into two groups, administered without (DM group) or with liraglutide (DM + Lira group) at a dose of 200 μg/kg/d. SD rats of control group were fed with normal chew and received subcutaneous injection of saline. All rats were anesthetized with sodium pentobarbital (60 mg/Kg body weight,ip) and sacrificed by exsanguinations at 20 weeks of age for the following experiments.

### Cell culture

The INS-1 rat insulinoma cell line was generously provided by Professor Cao Xiaopei (The First Affiliated Hospital, Sun Yat-sen University, Guangzhou). INS-1 cells were cultured in a humidified atmosphere containing 5% CO_2_ in complete medium (RPMI-1640 supplemented with 10% heat-inactivated fetal bovine serum, 1 mM sodium pyruvate, 50 μM 2-mercaptoethanol, 2 mM glutamine, 10 mM HEPES, 100 U/ml penicillin, and 100 μg/ml streptomycin). Cells were seeded at a density of 1×10^5^ cells/cm^2^ in 75-cm^2^ Corning bottles (Corning, USA) with 10 ml complete medium. The maintenance culture was passaged every 5–6 days by gentle trypsinization. For most experiments, unless otherwise mentioned, INS-1 cells were seeded at 1×10^5^ cells/cm^2^ in 2 ml media per well in 6-well plates (Corning, USA) and cultured for 5–6 days with medium change on day 3. A stock solution (100 nM) of palmitate (Sigma, St. Louis, MO) was prepared by dissolving the fatty acid in 50% ethanol and then diluted in culture medium with 0.5% fatty acid-free BSA (Sigma, St. Louis, MO) to a final concentration of 0.5 mM.

### miRNA microarray analysis

Total RNA was harvested using TRIzol (Invitrogen) and an miRNeasy mini kit (QIAGEN) according to the manufacturer’s instructions. After confirming RNA was of the required quantity and quality, the samples were labeled using the miRCURY™ Hy3™/Hy5™ Power labeling kit and hybridized on the miRCURY™ LNA Array (v.16.0). Following the washing steps, the slides were scanned using an Axon GenePix 4000B microarray scanner. Scanned images were then imported into GenePix Pro 6.0 software (Axon) for grid alignment and data extraction. Replicated miRNAs were averaged and miRNAs with intensities ≥ 30 in all samples were chosen for calculating the normalization factor. Expressed data were normalized using the median normalization method. After normalization, significant differences in miRNA expression were identified through volcano plot filtering. Finally, hierarchical clustering was performed with MEV software (v4.6, TIGR) to show distinguishable miRNA expression profiling among samples.

### qRT-PCR

qRT-PCR was performed using a commercial Kit (Takara, Japan) and an ABI7500 Real-time PCR system (Applied Biosystems, Carlsbad, USA). General principles of primer design were followed and complete genomic sequences of related RNA were searched for in the Genebank database. Total RNA was reverse transcribed with a Mir-X miRNA First-Strand Synthesis Kit and quantitative PCR was then performed with a Mir-X miRNA qRT-PCR SYBR Kit. The program was initially run for 2 min at 95°C, followed by 40 cycles of 30 sec at 95°C, and 60 sec at 60°C. The primer sequences were as follows: miR-139-5p forward primer, 5′-TCTACAGTGCACGTGTCTCCAG-3′, reverse primer, Uni-miR qPCR primer; U6 forward primer, 5′-ACGCAAATTCGTGAAGCGTT-3′, reverse primer, Uni-miR qPCR primer. Total RNA was reverse transcribed with an mRNA PrimeScript RT Master Mix Kit and quantitative PCR was then performed with an mRNA SYBR Premix Ex Taq II Mix Kit. The program was initially run for 2 min at 95°C, followed by 40 cycles of 30 sec at 95°C, and 60 sec at 60°C. The primer sequences were as follows: IRS1 forward primer, 5′-AAGCACCTATGCCAGCATCAAC-3′, reverse primer, 5′-GAGGATTGCTGAGGTCATTT AGGTC-3′; GAPDH forward primer, 5′-GGCACAGTCAAGGCTGAGAATG-3′, reverse primer, 5′-ATGGTGGTGAAGACG CCAGTA-3′, Data were extracted with the associated software of the ABI 7500 Real Time PCR System. The threshold cycle (Ct) values of the genes under examination in each sample were normalized using the values of the endogenous control mRNA (GAPDH) or miRNA (U6). The relative fold changes in mRNA or miRNA expression level were calculated with the 2^-ΔΔCt^ method.

### Immunohistochemistry

Formalin-fixed, paraffin-embedded pancreatic tissues were continuously sectioned into 3-μm-thick sections. The tissue sections were dewaxed in xylene, rehydrated, and rinsed in graded ethanol solutions. Antigen retrieval was performed by heating tissue sections at 100°C for 30 min in citrate (10 mmol/L, pH 6.0) or EDTA (1 mmol/L, pH 9.0) solution when necessary. The sections were then immersed in a 0.3% hydrogen peroxide solution for 30 min to block endogenous peroxidase activity, rinsed in phosphate-buffered saline for 5 min, and incubated with the primary antibody (mouse insulin mAb (Sigma, USA)) at 4°C overnight. Negative controls were prepared by replacing the primary antibody with a normal murine IgG antibody. The sections were then incubated with a horseradish peroxidase-labeled antibody directed against a rabbit secondary antibody (Sigma, USA) at room temperature for 30 min. Finally, the signal was developed for visualization with 3, 3′-diamino-benzidine tetrahydrochloride, and all slides were counterstained with hematoxylin. The color of the antibody staining in the tissue sections were observed by light microscopy.

### Detection of apoptosis

To identify apoptotic cells within pancreatic islets, the terminal deoxynucleotidyl transferase (TdT)-mediated dUTP end labeling (TUNEL) assay was performed on paraffin-embedded sections. The assay was performed according to the manufacturer’s instructions.

### Small RNA transfection

INS-1 cells were seeded in 6-well plates at a density of 1×10^5^ cells/well in 2 ml of supplemented RPMI-1640. After overnight incubation, cells were transfected with 50 nM of rno-miR-139-5p-mimics or their respective negative controls (RiboBio, Guangzhou, China) using Lipofectamine^TM^RNAMiX (Invitrogen) according to the manufacturer’s instructions. After 12 h, the transfection medium was replaced with serum-free DMEM. Total RNA or protein samples were extracted using TRIzol after 24 h (Invitrogen) or RIPA buffer after 48 h, respectively, from both transfected and untransfected INS-1 cells.

### 3′-UTR luciferase assay

The 3′-UTR luciferase assay was performed as previously described (Boon et al., 2011). Fragments of the 3′UTR of IRS1 containing putative or mutated miR-139-5p binding sites were amplified by RT-PCR using specific primers (S1 Table). According to the manufacturer’s instructions, the products were cloned into pmiR-RB-REPORT^TM^ vectors (Ribio Biotech, Guangzhou, China) downstream from the hRluc luciferase coding sequence. The constructs were cotransfected with miR-139-5p or a negative control (miR-139-5p-NC) into 293T cells using Lipofectamine 2000 (Invitrogen, USA). After 48 h of transfection, luciferase activity was measured using the Dual-Luciferase Reporter Assay System Kit (Promega Biotech, Madison, WI, USA). The activities of hRluc were first normalized to the internal control (hluc) to evaluate the transfection efficiency. All experiments were performed three times. MiR-139-5p mediated suppression of hRluc/hluc activity was calculated as the ratio of hRluc/hluc in miR-139-5p-transfected cells to hRluc/hluc in control oligotransfected cells.

### Western blot

Western blot analysis of IRS1, Bcl-2, and GAPDH were performed according to standard procedures. All antibodies were from Cell Signaling Technologies and diluted 1:1000 in 1% BSA. INS-1 cells and pancreatic tissue were lysed in RIPA buffer on ice and protein concentrations were determined using the Pierce BCA Protein Assay Kit (Thermo Scientific, Waltham, MA, USA). Equal amounts of protein (20 μg) were electrophoresed through 4% SDS-polyacrylamide stacking gels and 10% SDS-polyacrylamide separating gels and then transferred to PVDF membranes (Millipore, Darmstadt, Germany). After blocking at room temperature in TBST containing 5% BSA, membranes were incubated with primary antibodies against INS-1 and GAPDH overnight at 4°C with gentle shaking. Following three washes in TBST, membranes were incubated with secondary antibodies conjugated to horseradish peroxidase. Signal development was performed with Novex ECL (Invitrogen), films were scanned into TIFF format at 600 dpi resolution, and ImageJ software was used for signal quantification (Heinke et al., 2008).

### Statistical analysis

All experiments were performed in triplicate and data are presented as mean ± SEM. Comparisons between groups were by Student’s *t*-test using SPSS 16.0 statistical software (SPSS, Chicago, IL, USA). P<0.05 was considered statistically significant.

## Results

### Anti-apoptotic effect of liraglutide on pancreatic tissue of diabetic rats

**Fig 1:**
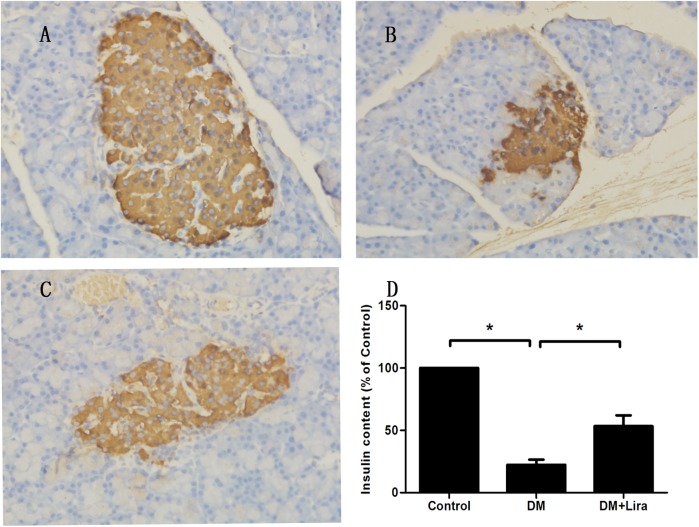
**Immunohistochemical staining of insulin from the control group (A), DM group (B), and DM + liraglutide group (C).** Islet-positive regions are brown. Fig 1D shows the relative insulin content of the three groups. Diabetic rats were divided into two groups, administered without (DM group) or with liraglutide (DM + Lira group) at a dose of 200 μg/kg/d. SD rats of control group were fed with normal chew and received subcutaneous injection of saline (Control group, DM group, and DM + liraglutide group; 100%, 22.3 ± 4.2%, 55.3 ± 8.7%, n = 5, p<0.05).

**Fig 2:**
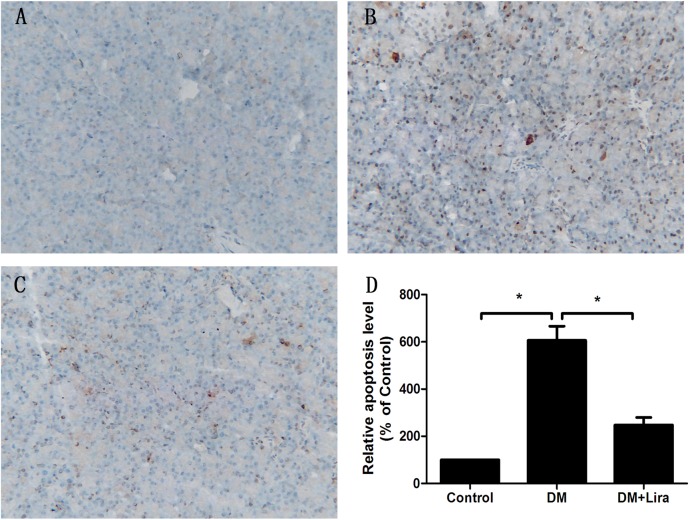
**Cell apoptosis of rat pancreatic tissue from the control group (A), DM group (B), and DM + liraglutide group (C) assessed by TUNEL staining.** TUNEL-positive regions are brown. Fig 2D shows the relative levels of apoptosis in the three groups. Diabetic rats were divided into two groups, administered without (DM group) or with liraglutide (DM + Lira group) at a dose of 200 μg/kg/d. SD rats of control group were fed with normal chew and received subcutaneous injection of saline (Control group, DM group, and DM + liraglutide group; 100%, 606.3 ± 59.9%, 247.7 ± 31.8%, n = 5, p<0.05).

**Fig 3:**
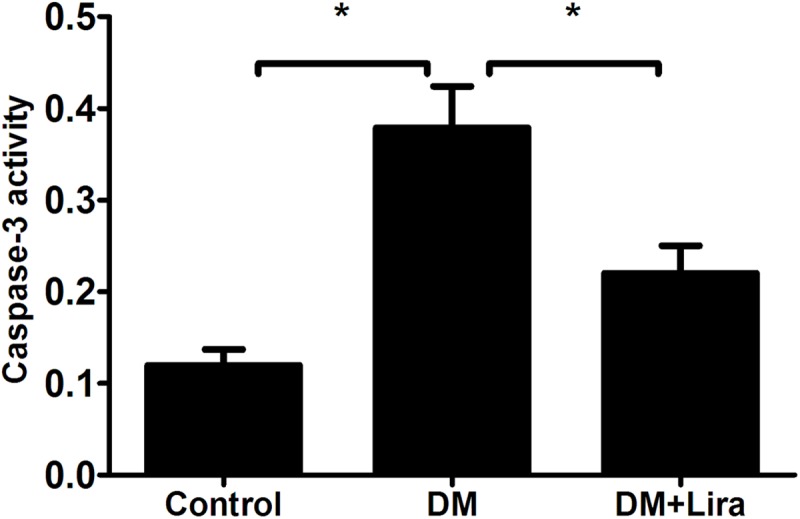
Caspase-3 activity was compared among the control group (A), DM group (B), and DM + liraglutide group (C) (n = 5,*p<0.05). Caspase-3 activity was decreased by 43.6% in the DM + liraglutide group compared with the DM group. Diabetic rats were divided into two groups, administered without (DM group) or with liraglutide (DM + Lira group) at a dose of 200 μg/kg/d. SD rats of control group were fed with normal chew and received subcutaneous injection of saline (DM group: 0.39 ± 0.10 vs. DM + liraglutide group: 0.22 ± 0.07) (p<0.05).

The degree of apoptosis in pancreatic tissue from diabetic rats treated with or without liraglutide was evaluated by immunohistochemistry, TUNEL assay, and detection of caspase-3 activity. We found that pancreatic tissue from diabetic rats showed aberrant upregulation of apoptosis compared with control groups, while apoptosis was downregulated in diabetic rats treated with liraglutide (p<0.05) (Figs 1 and 2). Caspase-3 activity was also compared among the three groups. As shown in Fig 3, caspase-3 activity was increased by 2.25 fold in the DM group compared with the control group (control group: 0.12 ± 0.04 vs. DM group: 0.39 ± 0.10). Furthermore, caspase-3 activity was decreased by 43.6% in the DM + liraglutide group compared with the DM group (DM + liraglutide group: 0.22 ± 0.07 vs. DM group: 0.39 ± 0.10) (p<0.05) (Fig 3). These data suggest that liraglutide protects against diabetes-induced toxicity by inhibiting apoptosis of pancreatic tissue.

### Liraglutide reduces miR-139-5p expression in pancreatic β-cells in vivo

**Fig 4:**
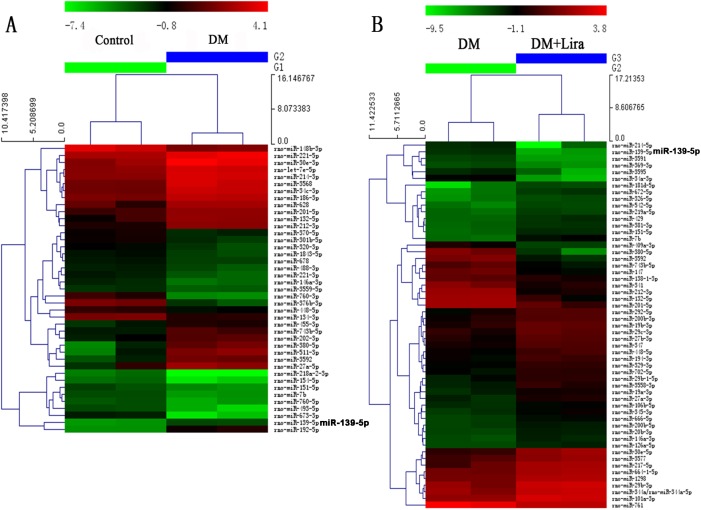
Cluster analysis of the microRNA expression levels in rat pancreatic tissues between the different groups. **(A) DM group vs. control group; (B) DM group vs. DM + liraglutide group).** Diabetic rats were divided into two groups, administered without (DM group) or with liraglutide (DM + Lira group) at a dose of 200 μg/kg/d. SD rats of control group were fed with normal chew and received subcutaneous injection of saline.

To survey the miRNAs expressed in pancreatic β-cells and determine their response to liraglutide, we profiled their expression levels using an miRCURY™ LNA Array (v.16.0) with samples derived from pancreatic tissue of the three groups of rats (Fig 4).

Compared with the control group, 20 miRNAs were differentially expressed in the DM group (20 increased by ≥ 2.0 fold with p<0.05). There were seven miRNAs reduced by over 50% (miR-380-5p, miR-139-5p, miR-743b-5p, miR-212-3p, miR-132-5p, miR-3592, and miR-201-5p) after liraglutide treatment. Among these, miR-139-5p expression increased by 4.1-fold in the DM group, and it decreased by over 90% following liraglutide treatment (Table 1).

**Table 1:** Differential expression of miRNAs in rat pancreatic tissues between the three groups (DM group, control group, and DM + liraglutide group).

miRNA species	DM vs. Control		DM + liraglutide vs. DM	
	Fold change	*P*	Fold change	*P*
rno-miR-380-5p	13.3411	0.0333	0.0331	0.0283
rno-miR-139-5p	4.0938	0.0057	0.0715	0.0039
rno-miR-3592	10.8368	0.0078	0.0863	0.0065
rno-miR-743b-5p	4.2472	0.0364	0.2292	0.0397
rno-miR-132-5p	3.7398	0.0203	0.1749	0.0084
rno-miR-212-3p	4.3228	0.0006	0.1684	0.0010
rno-miR-201-5p	2.9069	0.0019	0.3271	0.0207

**Fig 5:**
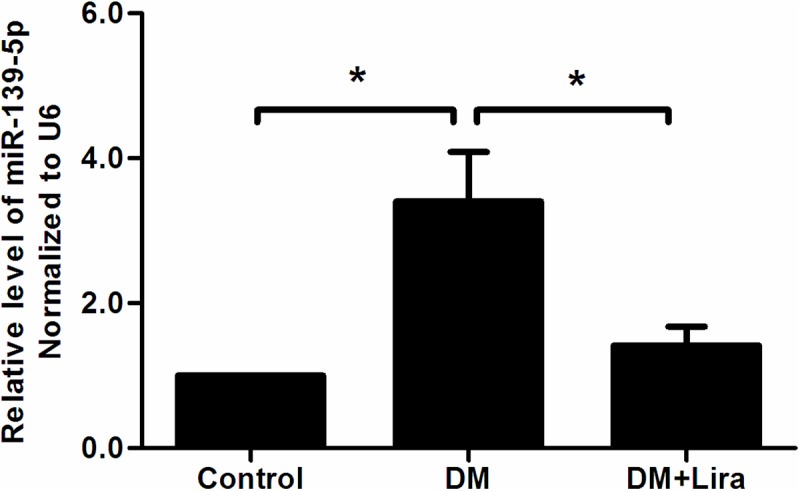
MiR-139-5p expression after liraglutide treatment by qRT-PCR (*p<0.05). (Control group, DM group, and DM + liraglutide group: 1.0, 3.4 ± 0.68, 1.4 ± 0.3, p<0.05). Diabetic rats were divided into two groups, administered without (DM group) or with liraglutide (DM + Lira group) at a dose of 200 μg/kg/d. SD rats of control group were fed with normal chew and received subcutaneous injection of saline.

Quantitative PCR analysis confirmed that miR-139-5p levels in the DM group were significantly higher than those in the control group (DM group: 3.4 ± 0.68 vs. control: 1.0, p<0.05). In contrast with the DM group, the expression levels of miR-139-5p decreased after liraglutide treatment (DM group: 3.4 ± 0.68 vs. DM + liraglutide:1.4 ± 0.3, p<0.05) (Fig 5). These data suggest that miR-139-5p may mediate the anti-apoptotic effect of liraglutide in pancreatic tissue.

### MiR-139-5p contributes to the anti-apoptotic effect of liraglutide in INS-1 cells

**Fig 6:**
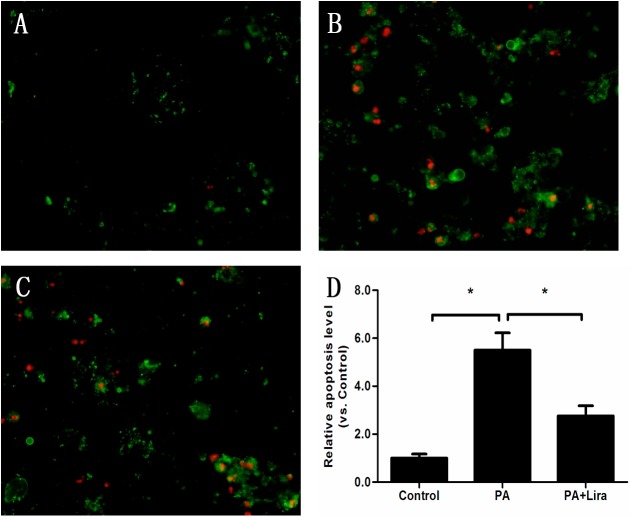
Annexin V and PI staining of INS-1 cells apoptosis treated with vehicel, palmitic acid (0.5 mM) and liraglutide (100 nM). Cells were treated with vehicle (A), palmitic acid (B) or palmitic acid + liraglutide (C) for 48 h, incubated with annexin V and PI, and analyzed by confocal microscope. Membranes of apoptotic cells were stained with annexin V and emitted bright green fluorescence. Nuclei of cells with damaged cell membranes were stained with PI and emitted red fluorescence. D: An estimate of the percentage of green-stained cells. Results are representative of three independent experiments (*p<0.05).

To determine the anti-apoptotic effect of liraglutide in INS-1 cells, INS-1 cells were costained with annexin V and propidium iodide (PI) (Fig 6). It has been previously reported that a sustained exposure to fatty acids impairs glucose-stimulated insulin secretion (GSIS), promotes apoptosis, and alters insulin gene expression[13, 14].Cells were treated with palmitic acid (0.5 mM) or palmitic acid + liraglutide (100 nM) for 48 h. Palmitic acid caused a significant 4-fold increase in apoptosis in INS-1 cells compared with cells transfected with vehicle (control) (palmitic acid group: 5.5 ± 0.7 vs. control group: 1.0, p<0.01). There were significantly less cells costained with annexin V and PI (PI-positive (red) and annexin V-positive (green)) in the liraglutide-treated group compared with palmitic acid group (palmitic acid+ liraglutide group: 2.8 ± 0.4 vs. palmitic acid group: 5.5 ± 0.7, p<0.01).

**Fig 7:**
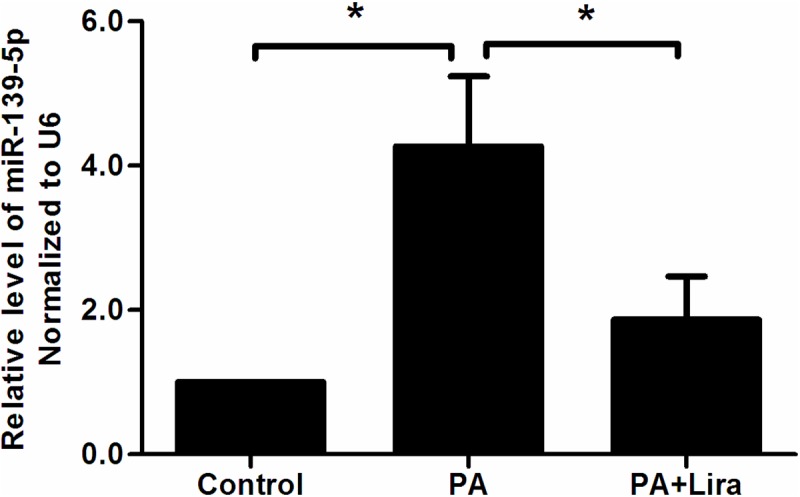
MiR-139-5p expression in INS-1 cells treated with palmitic acid (PA) and palmitic acid+ liraglutide (PA+Liar) (n = 3,*p<0.05). Cells were divided into control, PA and PA+Lira group, which respectively treated with vehicle, palmitic acid (0.5nmol/L) or palmitic acid (0.5mmol/L) + liraglutide (100nmol/L) for 48 h.

In INS-1 cells exposed to liraglutide, miR-139-5p expression was markedly downregulated compared with control cells exposed to palmitic acid (palmitic acid group: 4.3 ± 0.97 vs. palmitic acid+ liraglutide group: 1.9 ± 0.6) (n = 3, p<0.05) (Fig 7).

**Fig 8:**
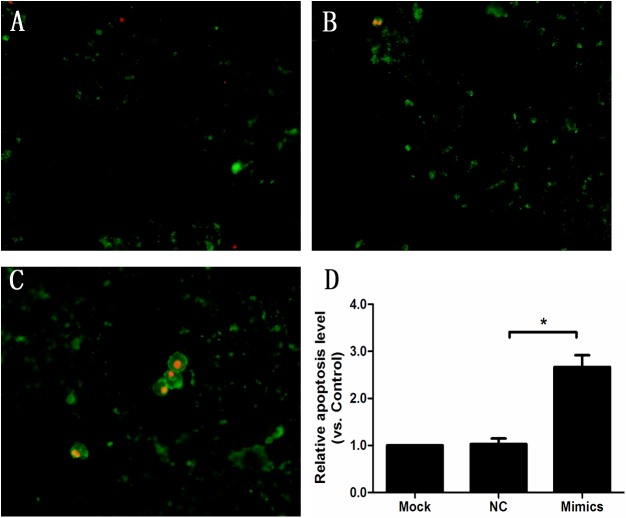
Overexpression of miR-139-5p enhanced INS-1 cell apoptosis by analysis with annexin V and PI staining (n = 3,*p<0.05). Cells were treated with negative control (B) or miR-139-5p mimics (C), incubated with annexin V and PI, and analyzed by confocal microscope. D: An estimate of the percentage of green-stained cells. Results are representative of three independent experiments. Cells were treated with Mock, NC (negative control) and Mimics for 24h. All experiments were performed in triplicate. Comparisons between groups were by Student’s t-test.

Overexpression of miR-139-5p increased apoptosis of INS-1 cells (Fig 8). Compared with control, there was a 1.7-fold increase of apoptotic cells in the miR-139-5p mimics-treated cells compared with the control cells as determined by annexin V and PI staining (control group: 1.0 vs. miR-139-5p mimics group: 2.7±0.3) (Fig 8D).

**Fig 9:**
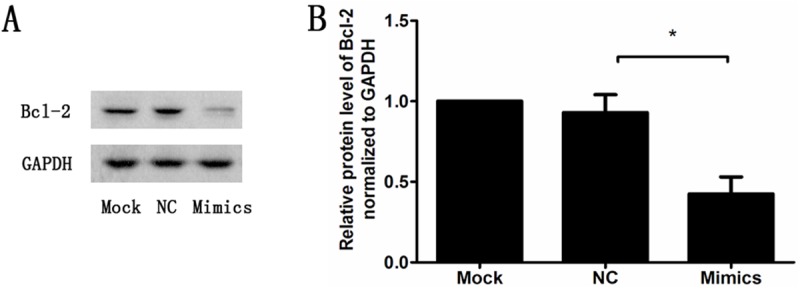
Bcl-2 expression was significantly decreased in miR-139-5p transfected INS1 cell model (n = 3,*p<0.05). Cells were treated with Mock, NC (negative control) and miR-139-5p Mimics for 24h.

We analysis the level of Bcl-2 in miR-139-5p transfected INS1 cell model (Fig 9). Bcl-2 expression was significantly decreased in the miR-139-5p mimics group compared with the Mock group (Mock group 1.0 vs mimics group 0.44, P<0.05). Our study suggest that miR-139-5p may promot apoptosis by targeting BCL2 in INS-1 cells. Caspase-3 activity was increased in the miR-139-5p mimics group compared with the Mock group (S1 Fig).

### MiR-139-5p specifically suppresses IRS1 expression

After a search of bioinformatics websites such as TargetScan, we found that miR-139-5p was complementary to the sequence, ACTGTAG, on the 3′-UTR of IRS1. Descriptions of the binding site of rno-miR-139-5p and the IRS1 gene are presented on the miRDB website.

**Fig 10:**
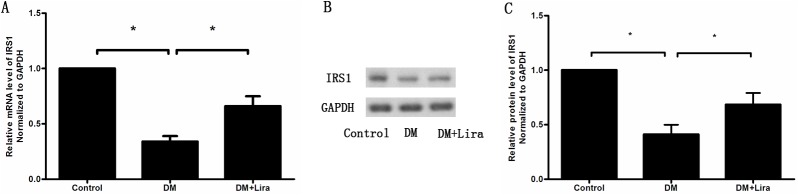
**mRNA and protein levels of IRS1 in pancreatic tissues were measured by qRT-PCR (A) and Western Blot (B and C).** Diabetic rats were divided into two groups, administered without (DM group) or with liraglutide (DM + Lira group) at a dose of 200 μg/kg/d. SD rats of control group were fed with normal chew.

We first assessed whether liraglutide can induce IRS1 expression in pancreatic tissue of diabetic rats. As shown in Fig 10A, the mRNA levels of IRS1 were significantly downregulated in the DM group and were upregulated following liraglutide treatment (DM group:0.34 ± 0.09 vs. DM+liraglutide group: 0.66 ± 0.16) (n = 3, p<0.05). The relative protein expression levels of IRS1 in the control group, DM group, and DM + liraglutide group were 1.0, 0.41 ± 0.16, and 0.68 ± 0.19, respectively (n = 3, p<0.05) (Fig 10B and 10C).

**Fig 11:**
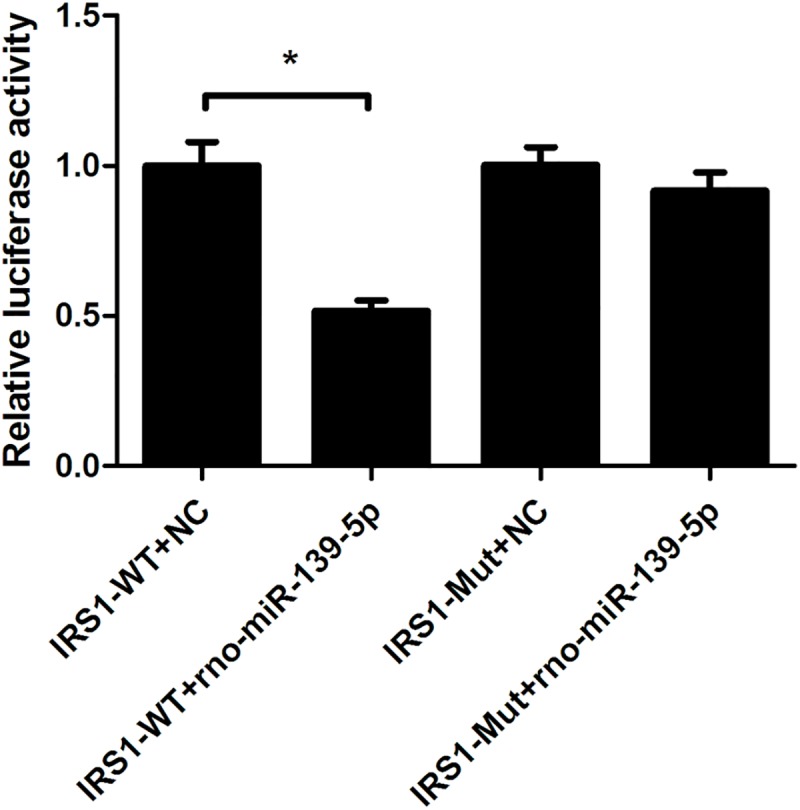
Dual-luciferase reporter assay of IRS1 and rno-miR-139-5p (n = 3, p<0.05). Cells were cotransfected with the luciferase reporter vector containing wild-type (IRS1-WT) or mutated (IRS1-Mut) 3’-UTR of IRS1 and miR-139-5p mimic (miR-139-5p) or control oligonucleotides (NC). Data are shown as means ± SD of three independent experiments. It demonstrated that miR-139-5p inhibited the IRS1 expression by interacting with the 3’-UTR of IRS1.

We then performed a dual-luciferase reporter assay of IRS1. The relative luciferase activity of the negative control group with wild-type carriers was 1.00 ± 0.08. It was 0.52 ± 0.04 and 1.00 ± 0.06 for the rno-miR-139-5p group with wild-type carriers and the negative control group with mutant carriers, respectively. Regarding the rno-miR-139-5p group with mutant carriers, the relative activity was 0.92 ± 0.06 (Fig 11). These data suggest that rno-miR-139-5p may specifically regulate IRS1 expression by targeting ACTGTAG at the 3′-UTR.

**Fig 12:**
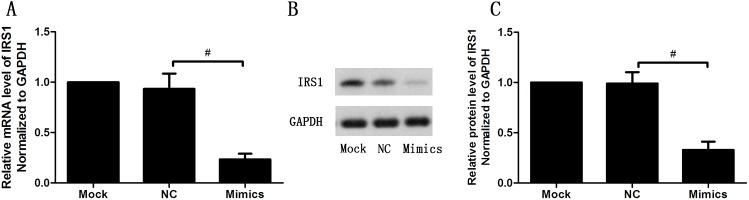
**Effect of transfection with miR-139-5p mimics on IRS1 expression level in INS-1 cells measured by qRT-PCR (A) and Western Blot (B and C) (n = 3,#p<0.05).** Cells were treated with Mock, NC (negative control) and Mimics for 24h. All experiments were performed in triplicate. Comparisons between groups were by Student’s t-test.

We next studied the effects of transfection with miR-139-5p mimics on IRS1 expression level in INS-1 cells. After transfection with miR-139-5p mimics, IRS1 mRNA decreased by 70% compared with the negative control (NC) group (p<0.05). The relative mRNA levels of miR-139-5p in mock group, NC group and mimics group were 1.0, 0.93 ± 0.15, and 0.23 ± 0.06. Regarding IRS1 protein, the relative levels were 1.0, 0.99 ± 0.11, and 0.33 ± 0.08 (p<0.05) for the mock, NC, and mimics group (Fig 12). These data indicate that transfection with miR-139-5p mimics can suppress IRS1 expression in INS-1 cells.

## Discussion

In the present study, we sought to identify specific miRNAs that may mediate the action of GLP-1 on β-cell apoptosis. Induction of miR-139-5p was observed in diabetic rats and INS-1 cells treated with liraglutide. The overexpression of miR-139-5p increased INS-1 cell apoptosis. Mechanistically, we demonstrated that miR-139-5p specifically suppressed IRS1 expression. Our findings suggest a new pathway that links GLP-1 to inhibition of β-cell apoptosis by downregulating the targeting of miR-139-5p to IRS1.

Previous studies have suggested that the GLP-1 receptor expressions in pancreatic islets[15]. The protective effects of GLP-1 analogs against glucotoxicity and/or lipotoxicity have previously been investigated in vitro in INS-1 and MIN6 cells and in vivo in HFD and STZ mice [3]. For example, liraglutide decreased HFD-induced β-cell apoptosis by activation of PI3K/Akt, which resulted in inactivation of FoxO1 along with the downregulation of p27, Bax, and Cidea and upregulation of Pdx-1, MafA, and NeuroD [16].

Recent studies reported that the signaling pathways triggered by GLP-1 in rodent and human pancreatic β-cells were complex, with miRNA-132 and miR-212 as two of the many miRNAs involved in GLP-1 activity. The inductions of miR-132 and miR-212 by GLP-1 were correlated with cyclic adenosine monophosphate (cAMP) production and were blocked by a protein kinase A inhibitor[17]. Overexpression of miRNA-132 or miRNA-212 increased glucose-stimulated insulin secretion [17]. We therefore hypothesized that miRNAs might be important in liraglutide-induced β-cell protection against lipid stress. To address this question, we selected miR-139-5p, which is differentially expressed in the pancreatic tissue of liraglutide-treated rats compared with DM and control rats, based on gene-chip microarray analysis and confirmed by qRT-PCR. The expression of miR-139-5p demonstrated several-fold increases in DM rats and INS-1 cells (data not shown) and was reduced by liraglutide treatment. GLP-1 and miR-139-5p both target cells apoptosis, but their potential links were unclear. The findings reported in this manuscript reveal that expression of miR-139-5p decreased after GLP-1 treatment in both cellular and animal models. Therefore, GLP-1 may play an important role in biology by downregulating miR-139, which introduces a new research direction for the treatment of diabetes using GLP-1. Whether liraglutide inhibited miR-139 via pathways such as cAMP/protein kinase A-dependent pathway need to be investigated in future studies.

MiRNA networks represent control points for integration of environmental and genetic factors that influence the physiological behavior of β-cells [18]. The potential participation of miRNAs in palmitate-induced lipoapoptosis was not investigated until recently. It was reported that miR-34a promotes palmitate-induced apoptosis in INS-1 cells, and the downregulation of miR-34a by GLP-1 alleviated the lipotoxicity caused by palmitate [19]. Upregulation of miR-34a-5p and the associated inhibition of Bcl-2/Bcl-w are involved in mediating stearic acid cytotoxicity [20]. Although miR-139-5p has been described in a limited number of studies as a potential tumor suppressor miRNA [21, 22], little is known about the function or regulation of miR-139-5p in β-cells. The findings of the present study demonstrated three aspects of this highly regulated miRNA species, which is likely a critical mediator of β-cell apoptosis. First, we identified miR-139-5p as a novel miRNA that is regulated by palmitic acid and liraglutide. Second, overexpression of miR-139-5p enhanced INS-1 cell apoptosis, associated with caspase-3 activity and Bcl-2 expression. Third, we found that miR-139-5p is a key intermediary that links GLP-1 signaling with suppression of IRS1 expression. MiR-139-5p has been reported to promoting apoptosis by targeting c-Met and BCL2 in cancer cells [12, 23]. Our study suggest that miR-139-5p may promot apoptosis by targeting BCL2 in INS-1 cells. Whether miR-139-5p directly targets Bcl-2 within the Bcl-2 3′-UTR in the luciferase reporter analysis need to be further investigated. Since miRNAs may have species-specific targets, studies on isolated rat and human islets require further investigation.

IRS1 plays a key role in transmitting signals from the insulin and insulin-like growth factor-1 (IGF-1) receptors to intracellular pathways such as the PI3K/Akt pathway and mitogen-activated protein (MAP) kinase pathway [24]. Reduced tyrosine phosphorylation of the IRS1 protein contributes to peripheral insulin resistance and β-cell failure [25, 26]. We found that palmitic acid can increase the expression of miR-139-5p and reduce IRS1 expression at both the mRNA and protein level. We performed a dual-luciferase reporter assay and found that rno-miR-139-5p may specifically regulate IRS1 expression by targeting ACTGTAG at the 3′UTR. Additionally, transfection with miR-139-5p mimics suppressed IRS1 expression in INS-1 cells. These findings suggest that increases in miR-139-5p expression increase β-cell apoptosis by targeting IRS1. However, the mechanisms by which downstream protein kinases promote β-cell apoptosis require further investigation, given that IRS1 contains over 70 potential serine/threonine residues for several protein kinases, including casein kinase II, cAMP-dependent protein kinase, protein kinase C, cell division cycle 2 kinase, MAP kinase, and protein kinase B/Akt[27]. Our results are supported by two recent findings. One is that dipeptidyl peptidase-4 inhibition improves β-cell function in db/db mice, but not in any of the IRS1-deficient mouse strains [28]. The other is that MicroRNA-139-5p suppresses 3T3-L1 preadipocyte differentiation through Notch and IRS1/PI3K/Akt insulin signaling pathways[29]. Whether inhibition of IRS1 can promote the effects of miR-139-5p on apoptosis of INS-1 cells needs to be further investigated.

In conclusion, our study confirmed that 1) elevated levels of miR-139-5p in the pancreatic tissues of diabetic rats can suppress IRS1 gene expression to promote cell apoptosis, and 2) liraglutide has anti-apoptotic effects by downregulating miR-139-5p levels and upregulating IRS1 gene expression. These findings provide key mechanistic insights into the critical miRNA-based crosstalk between two GLP-1 signaling cascades that regulate important early steps in β-cell apoptosis.

## Supporting information

S1 Fig Caspase-3 activity was increased in the miR-139-5p mimics group compared with the NC group (n = 3,*p<0.05). Cells were treated with Mock, NC (negative control) and miR-139-5p Mimics for 24h.
(TIF)

S1 Table RT-PCR primer sequences of IRS1 in the 3′-UTR luciferase assay. (DOCX)
