# Supplementary figures and images for: Downregulation of miR-139-5p contributes to the antiapoptotic effect of liraglutide on the diabetic rat pancreas and INS-1 cells by targeting IRS1
Source: PLoS One. 2017 Mar 27;12(3):e0173576. doi: 10.1371/journal.pone.0173576 (PMC5367678; doi:10.1371/journal.pone.0173576)

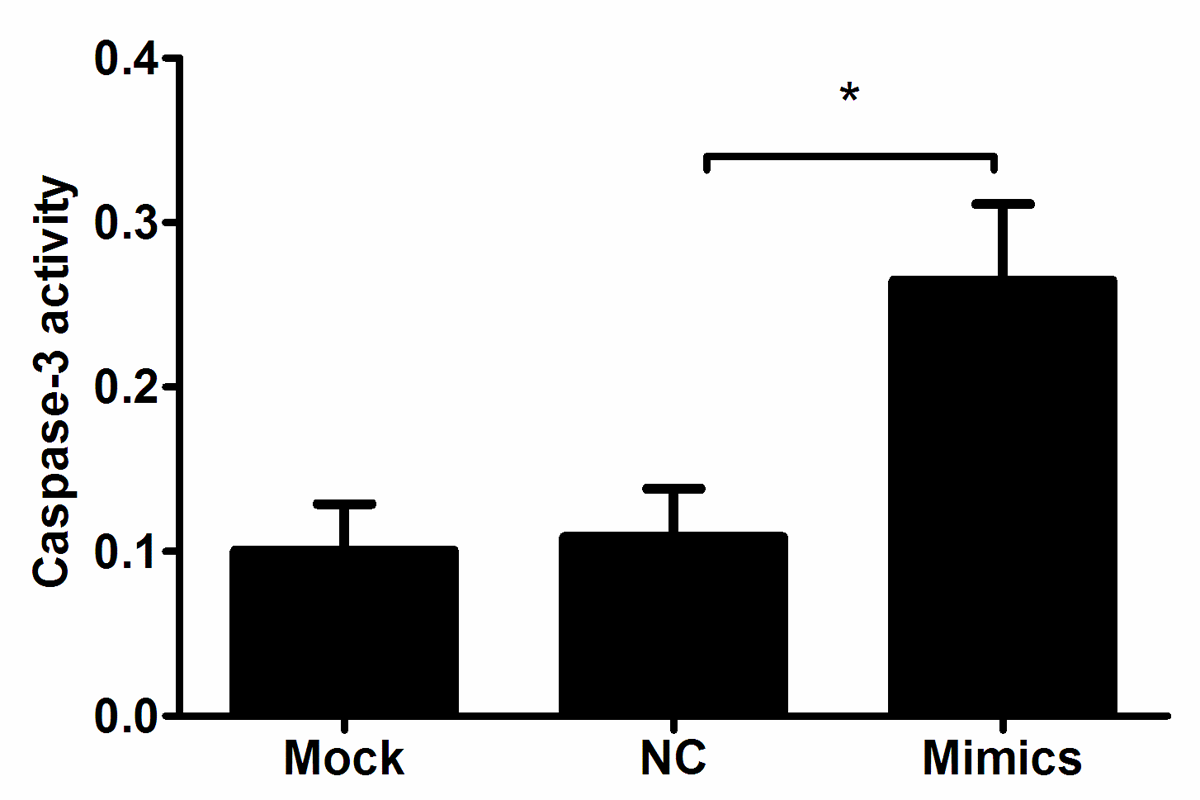

Supplement: S1 Fig — Cells were treated with Mock, NC (negative control) and miR-139-5p Mimics for 24h. (TIF) [file pone.0173576.s001.tif]
