# Supplementary material for: Downregulation of miR-139-5p contributes to the antiapoptotic effect of liraglutide on the diabetic rat pancreas and INS-1 cells by targeting IRS1
Source: PLoS One. 2017 Mar 27;12(3):e0173576. doi: 10.1371/journal.pone.0173576 (PMC5367678; doi:10.1371/journal.pone.0173576)
Supplement: S1 Table — (DOCX) [file pone.0173576.s002.docx]

S1 Table RT-PCR primer sequences of IRS1 in the 3′-UTR luciferase assay

| Primers | Sequences (5 'to 3') |
| --- | --- |
| r_Irs1_3’UTR_F | AATCTCGAGCTTAACTGGACGTCACAGCAGAATGAAGACCTAAATGACCTCAG |
| r_Irs1_3’UTR_R | AATGCGGCCG*C*GGGGGAAAGGCTTATAGAAG |
| r_Irs1_mut_F | TTGTAAATTGACATCAGAGAGACATGAGCGATC |
| r_Irs1_mut_R | GTCTCTCTGATGTCAATTTACAATGGAAGTCTG |
